# Prediction of post-treatment recurrence in early-stage breast cancer using deep-learning with mid-infrared chemical histopathological imaging

**DOI:** 10.1038/s41698-024-00772-x

**Published:** 2025-01-17

**Authors:** Abigail Keogan, Thi Nguyet Que Nguyen, Pascaline Bouzy, Nicholas Stone, Karin Jirstrom, Arman Rahman, William M. Gallagher, Aidan D. Meade

**Affiliations:** 1 Radiation and Environmental Science Centre, Physical to Life Sciences Research Hub, Technological University Dublin, Dublin, Ireland; 2 School of Physics, Clinical and Optometric Sciences, Technological University Dublin, City Campus, Dublin, Ireland; 3 Digital Futures Research Hub, Technological University Dublin, Dublin, Ireland; 4 Department of Physics and Astronomy, University of Exeter, Exeter, UK; 5 Division of Oncology and Therapeutic Pathology, Department of Clinical Sciences, Lund University, Lund, Sweden; 6 UCD School of Biomolecular and Biomedical Science, UCD Conway Institute, University College Dublin, Dublin, Ireland; 7 UCD School of Medicine, UCD Conway Institute, University College Dublin, Dublin, Ireland

**Keywords:** Breast cancer, Biophysics, Mathematics and computing

## Abstract

Predicting long-term recurrence of disease in breast cancer (BC) patients remains a significant challenge for patients with early stage disease who are at low to intermediate risk of relapse as determined using current clinical tools. Prognostic assays which utilize bulk transcriptomics ignore the spatial context of the cellular material and are, therefore, of limited value in the development of mechanistic models. In this study, Fourier-transform infrared (FTIR) chemical images of BC tissue were used to train deep learning models to predict future disease recurrence. A number of deep learning models were employed, with champion models employing two-dimensional and two-dimensional-separable convolutional networks found to have predictive performance of a ROC AUC of approximately 0.64, which compares well to other clinically used prognostic assays in this space. All-digital chemical imaging may therefore provide a label-free platform for histopathological prognosis in breast cancer, opening new horizons for future deployment of these technologies.

## Introduction

Approximately 2.2 million women worldwide were diagnosed with breast cancer in 2020, rendering it the most frequently occurring cancer in women^1^. Of these, approximately 80% are hormone receptor positive, and therefore candidates for cytotoxic adjuvant chemotherapy; the clinical decision to undergo this type of therapy is influenced by the risk profile of each patient^2^. Human epidermal growth factor receptor 2 (HER2)-negative, hormone receptor positive patients with early disease may have their risk profile determined by a range of clinicopathological risk factors including patient age, tumor size and grade, and lymph node (LN) involvement. The Nottingham Prognostic Index (NPI)^3,4^, which combines tumor size, grade and LN involvement, classifies early and locally advanced breast cancer cases into three or more prognostic groups.

For early-stage ER+ LN− patients, several tests are available on the market that attempt to stratify based on the likelihood of future disease recurrence. These include but are not limited to, the Oncotype DX test^5^ (a qRT-PCR assay measures the gene expression levels of 16 cancer-related genes plus 5 control genes) and the MammaPrint test^6^ (which uses a 70 gene profile). Another test, OncoMasTR, has recently emerged that calculates a Molecular Score (OMm), which contains information relating to solely 3 master transcription regulators (MTRs) and 3 reference genes, and its composite OncoMasTR Risk Score (OM), which combines OMm with LN involvement and tumor size and categorizes patients as having either low or high risk of recurrence. This test has been analytically validated^7^ and clinically validated to Level 1 evidence via two category B studies as significantly prognostic for breast cancer recurrence in two independent patient cohorts^8,9^.

Despite the clinical use and development of next-generation transcriptomic assays to predict breast cancer treatment outcome, discordance in the risk classification of individual patients with different tests confounds the choice of an optimal molecular-based signature for a given patient^10^. Moreover, despite their differential approaches to prognose treatment outcome, each test has a very similar predictive power, with OncoTypeDX having a ROC AUC of 0.69 and MammaPrint 0.59^11,12^. This indicates that each test, while powerful, also fails to identify a significant proportion of patients in whom cancer recurs. Additionally, tests such as OncoType-DX are expensive (~$3000 per patient in the UK) and have a 7-10 day turnaround time.

Aside from molecular tests, tumor organization is a key factor for disease progression, affecting the ability of the tumor to interact with the microenvironment^13,14^. Recent evidence suggests that robust predictive assays for recurrence which employ machine learning and artificial intelligence on haematoxylin and eosin stained pathological images have a performance approaching that achieved with genomic and proteomic tests^15^. Chemical imaging (via either Raman or FTIR microspectroscopy) offers an alternative, label-free, non-destructive approach to histopathological image generation which profiles histopathological tissue in both a spatial and biochemical context, with demonstrated applications both in the pathology laboratory^16^ and in surgery^17,18^, owing to its potential to image both preserved and fresh tissues. As a result it has seen rapid development towards devices for diagnosis of cancer and pre-cancerous lesions^19^, with some examples of the development of clinical support tools for diagnostic histopathology^20^, and more recently moves towards the development of prognostic tests^13^. FTIR imaging spatially captures a combined spectrum of the vibrations of molecular moieties including –NH, –C=O, –CN, –CH_3_, –CH_2_, and –PO_2_^21^. It has been demonstrated that spectral data are altered as a result of pre-translational (DNA methylation) and post-translational effects (e.g., phosphorylation, acetylation, glycosylation, etc.) in disease states^22,23^, such that objective histopathological classification models may be constructed. Recent evidence has also highlighted the connectivity between chemical imaging data and deep spatial tissue biology^24^. Crucially, analysis pipelines for chemical imaging data typically require the application of segmentation and dimensionality reduction steps before application of ML techniques, which may result in the loss of important spectral and spatial information, and therefore a pivot towards whole image analytical pipelines for this data is required.

The advent of deep-learning (DL) now offers a pipeline directly from image acquisition to classification either via digital pathology or whole image classification^25^. DL is an application of neural networks with many hidden layers that internally identify patterns in data without the need to recourse to feature engineering^26,27^. DL-convolution neural networks (CNNs) have demonstrated astounding abilities to resolve class across many computer vision tasks, including recognition of skin cancer subtype with simple RGB image data with performance exceeding that of Board accredited clinicians^28^. Essentially DL-CNNs may be presented with raw data and automatically discover features or their combinations which can learn, classify or detect objects within them^26,27^. Despite these advances, the development of network learning approaches specifically designed to work with chemical or hyperspectral imaging data, comprising of hundreds to thousands of channels measured spatially, is in its infancy^29^.

In the present study, an FTIR chemical-imaging approach to segregation of early stage BC patients according to downstream recurrence (or not) has been developed in a mixed cohort of patients with varying ER, PR and HER2 status. The study represents the first of its kind to use whole-image deep-learning pipelines for development of recurrence-segregation models with chemical imaging data. Several ML/DL pipelines employing both traditional algorithms and DL approaches were developed to model the connection between chemical images and all breast cancer recurrence (BCR) events (i.e., both local and distant). We demonstrate that chemical-imaging deep learning pipeline was found to have a prognostic performance ROC AUC of approximately 0.64 for prediction of BCR, with additional results demonstrating network capability in predicting other treatment endpoints such as BCR survival (up to 7 years post therapy). This BCR prediction performance agrees excellently with current transcriptomic tests for recurrence of this disease, pointing to the potential wider opportunities for deployment of this technology in the future.

## Methods

### Cohort characteristics and survival analysis

This study used formalin-fixed paraffin preserved tissue micro-array specimens from a consecutive Swedish cohort, which is described elsewhere^30,31^. A total of 1–2 tissue cores were taken from full-face tissue sections from 144 BC patients with invasive breast cancer who were diagnosed at Malmo University Hospital, Malmo, Sweden. Patients within this cohort did not receive neoadjuvant therapy and were treated with radical local mastectomy or wide local excision^32^.

Full details on the cohort characteristics are shown in Table 1 (nodal status was known for 130 patients). Due to extensive core loss, image data from a number of patients were excluded from analysis (5 of the ER+ patients, 3 HER2+).

**Table 1:** Cohort characteristics including endocrine receptor (ER), progesterone receptor (PR), human epidermal growth factor receptor (HER2) positivity, together with lymph node (LN) status, recurrence free survival (RFS) and overall survival

Characteristic	Value
Age at diagnosis (years)	65 (13.5)
ER+/−	125/19
PR+/−	100/44
HER2+/−	51/93
LN+/−	56/74
RFS (years)	5.45 (2.2)
OS (years)	5.78 (1.88)
NHG (1/2/3)	22/64/57
Therapy type (radiotherapy/chemotherapy/endocrine therapy)	60/113/96

Tumor grade is encapsulated as Nottingham Histological Grade (NHG) score.

In total, after imaging, a dataset from 139 patients was used for machine learning analysis, with 29 experiencing future recurrence of the disease (all but one of which were ER+).

**Fig. 1:**
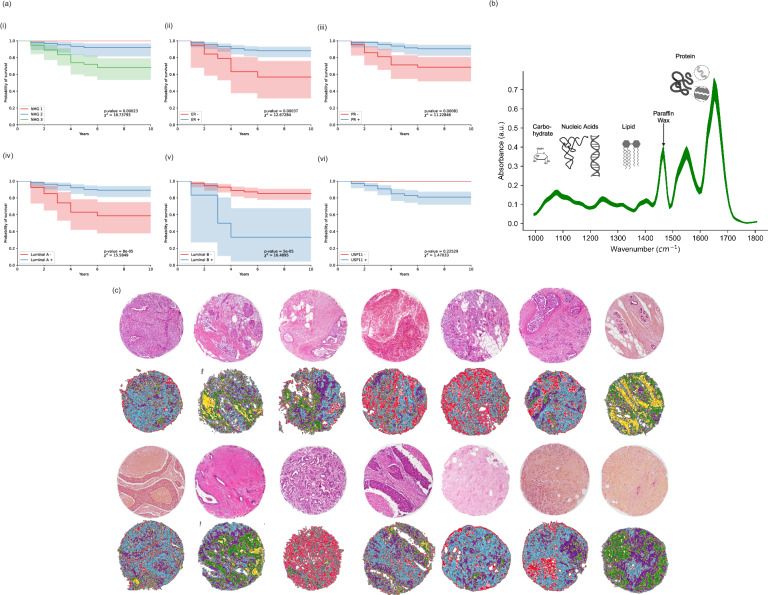
Cohort survival analysis, with introduction to Fourier-Transform infrared chemical imaging for histopathology. **a** Kaplan–Meier survival analysis for a selection of histological, physiological and molecular markers within the cohort, including (i) Nottingham histological grade, (ii) estrogen receptor status (iii) progesterone receptor status (iv) luminal type A, (v) luminal type B and USP11 positivity. **b** Sample FTIR spectra of BC tissue, with important bands of interest annotated biochemically. **c** Collage of haematoxylin-eosin stained images of TMA cores with matched FTIR chemical images of corresponding serial, unstained section (First and third row—H&E stained cores; second and fourth rows—serial chemical image). FTIR chemical images are false colored on the basis of a double application hierarchical auto-k-means clustering (with *k* varying from 2 to 5). This method employed the Pakhira-Bandyopadhay-Maulik index for optimization of the number of clusters across all FTIR images acquired in the study.

Kaplan–Meier survival analysis was conducted on the expression levels for a set of hormone receptors and molecular markers which were available for the cohort. This analysis was conducted in Python v. 3.9 and employed the lifelines package (v.0.29.0). The log-rank test was used for the calculation of chi-squared and *p* values. A visualization of the survival functions with respect to a selection of targets is provided in Fig. 1a.

### FTIR chemical imaging

A 5 µm thick slice of each TMA block was taken for FTIR imaging, with two contiguous sections taken for haematoxylin-eosin and immunohistochemical staining.

FTIR imaging was conducted using an Agilent Cary 620 IR microscope coupled with an Agilent Cary 670 FTIR spectrometer at the University of Exeter. This instrument uses a 128 × 128 pixel focal plane array, with 2 × 2 mosaic tiles capturing the full TMA core (typically 750 mm diameter). Spectra were collected at a spectral resolution of 16 cm^−1^ at 4 scans per pixel over the fingerprint region from 1000–1800 cm^−1^, where the number of scans was chosen to optimize the signal-to-noise ratio. A total of 245 images were selected to form the dataset in the study.

All samples were imaged without chemical dewaxation. Samples were excluded based on visual examination during collection; more intrusive collections of paraffin wax on the tissue resulted in exclusion and those with especially sparse tissue cores were not imaged. A sample FTIR spectrum of the tissue measured in this work is presented in Fig. 1b.

### FTIR image and spectral preprocessing

**Fig. 2:**
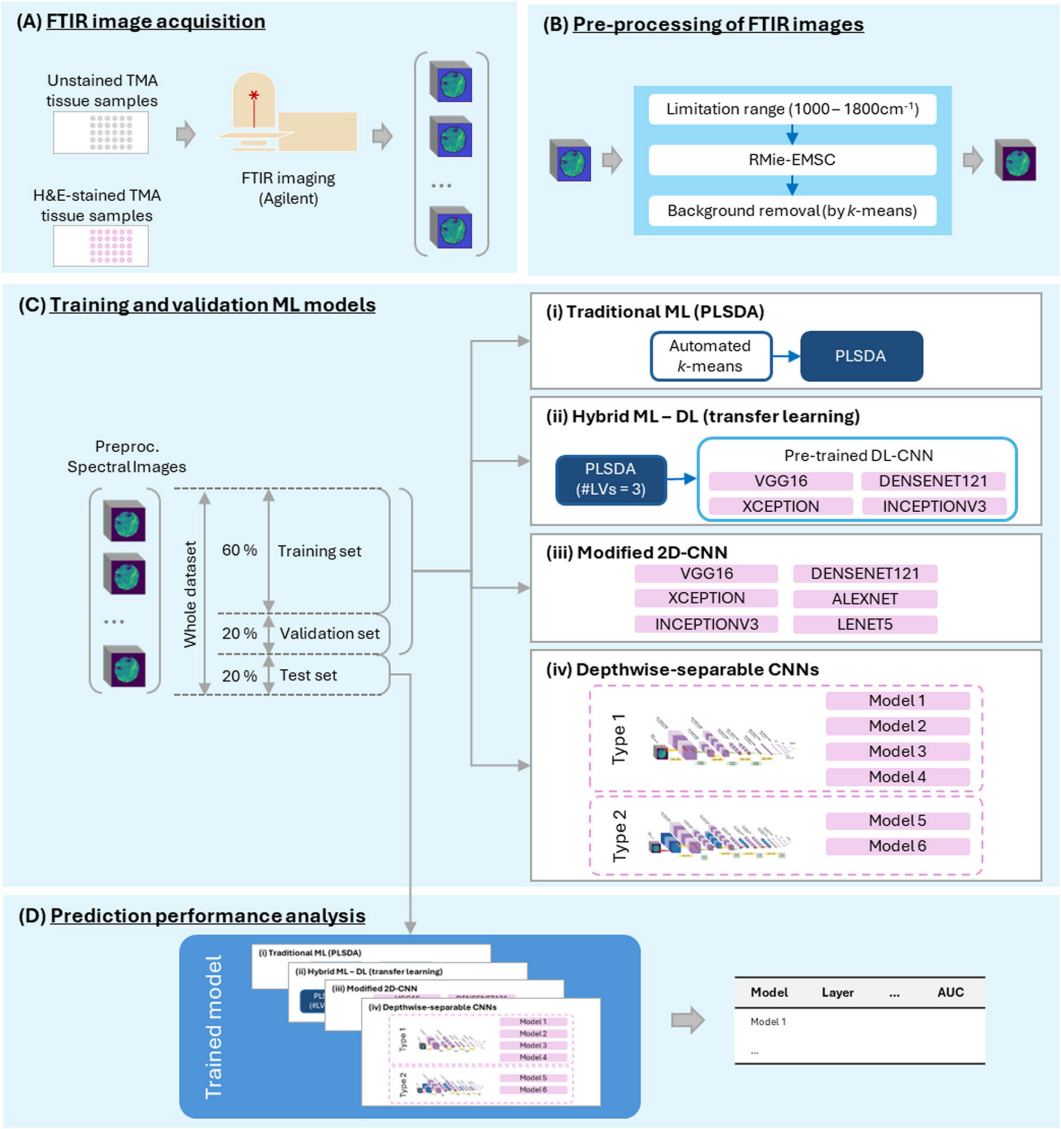
Visualization of data acquisition to modeling pipelines employed within the study. In (**A**), Fourier Transform Infrared (FTIR) imaging data were acquired from unstained TMA sections using a serial H&E section as ground truth. In (**B**), image data were preprocessed to truncate the spectral region to the fingerprint region, then to remove a spectral background and finally, to remove pixels surrounding the tissue which did not contain tissue spectra. In (**C**), the set of ML + DL modeling approaches for the prediction of recurrence are detailed, with prediction performance evaluated across all modelling approaches using the receiver-operator-characteristic area-under-the curve (**D**).

Prior to using FTIR spectral data for analysis, various pre-processing steps were implemented as shown in Fig. 2B. Specifically, the spectral baseline contribution from resonant Mie scattering was removed using a single iteration of the resonant Mie scattering-EMSC algorithm employing Matrigel as the reference spectrum^33–35^. This step was conducted in Matlab (R2021b, the Mathworks Inc.), with all subsequent processing and modeling steps conducted in Python 3.9, with sci-kit learn v.1.0 and tensorflow v.2.8.

Detection of pixels from the slide substrate around the image of the TMA core was performed using a *k*-means cluster algorithm with *k* = 2, with the image array padded with zeros for those pixels in which no tissue was present. A collage of spectral images from the TMA are provided in Fig. 1c, together with images of haematoxylin-eosin stained tissue sections which were cut contiguously to the section for spectral imaging, and which are paired with each corresponding spectral image. False-colored spectral images were generated from the application of crisp-cluster validity indices as described previously^36,37^.

### ML and DL pipeline development

The following represents a summary of the ML and DL strategies used in the study. Figure 2C, D provides a pictorial visualization of the set of ML and DL strategies employed in this study. For each individual model and modeling strategy training was conducted using images randomly selected from 60% of the patient cohort, validation with 20% and the remaining 20% held out for testing. A number of complete training-validation-testing rounds, within which image data were randomly sorted between training, validation and testing sets, was conducted for each model to assess model robustness. The sections following detail the approach in respect of each model type.

**Fig. 3:**
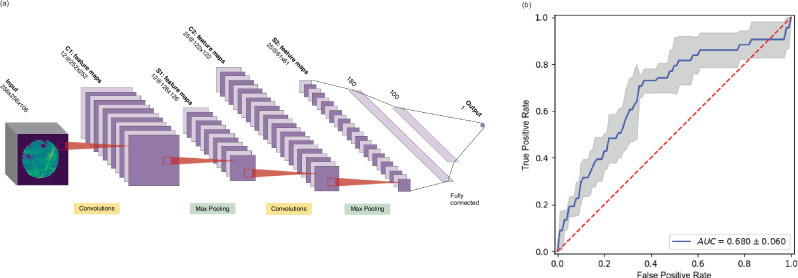
Modified LeNet5 deep learning model architecture with performance in predicting BC survival post-therapy. **a** Modified LeNet deep learning architecture for binary classification with chemical imaging data. **b** Typical performance of the model in (**a**) in predicting BC survival post therapy. Variability in performance is depicted as the shaded area around the curve.
- In the final approach, two avenues for deeper mining of spectral information of approaches were analyzed, involving (i) the implementation of 3D CNN layers^47^ and (ii) the implementation of depthwise separable CNN (DSCNN) layers^41,48^ (Fig. 2C (iv)).

To mine information from the spatial and spectral dimensions, one significant obstacle is the sheer size of each chemical image in data terms. As each image has an average size of 25 Mb, RAM drains quickly during training of ML and DL algorithms, even in scenario’s where high memory and parallelization of execution are available. Of course, the high dimensionality of the samples also complicates the learning process for a ML algorithm. As a consequence in the present work we have adopted several approaches to the implementation and training of ML models, limited by the aforementioned factors, for prediction of future recurrence with chemical imaging data:

- The first was to apply a traditional ML approach to prediction of recurrence with chemical imaging data^36^. Here images were segmented using an automated hierarchical double *k*-Means clustering algorithm which employed crisp cluster validity indices and which was applied across the entire dataset employ with *k*_min_ = 2 and *k*_max_ = 5^38,39^. Subsequently spectra from each cluster were used to train individual partial-least squares discriminant analysis (PLSDA) models for the predictive task (Fig. 2C (i)) with the PLSDA models built using 1–50 latent variables, and the optimal model complexity found for each iteration using an F1-score on the validation set. This process was repeated in its entirety on 10 occasions.
- The second was to employ a hybrid ML–DL transfer learning approach, where freely available 2-dimensional DL- CNN network architectures (VGG16^40^, Xception^41^, InceptionV3^42^, and DenseNet121^43,44^) were employed. For compression of the spectral dimension of these images to 3 channel variables a PLSDA model with 3 latent variables was built across a training set of spectra in the image dataset and the optimal model at training was used to construct a secondary image dataset. The PLS embeddings for each spectrum were resolved spatially such that each pixel of the image data was made up of the three PLS scores for each spectrum at that point. The test set was transformed using this model. The 3-channel image dataset was then passed through the early layers of pre-trained deep learning networks listed previously, with 2 fully connected layers added at the end for classification of recurrence (Fig. 2C (ii)).
- In the third approach the networks in (b) were modified to allow input of raw, hyperspectral, chemical imaging data for binary classification, and were trained from scratch. This approach and approach (b) investigated the hypothesis that coupling spectral information to spatial information would provide improved predictive performance (Fig. 2C (iii)). Full FTIR spectral images were fed directly into networks with architectures similar to well-known networks including those listed in (b) above, and both AlexNet^45^ and LeNet5^46^. The initial layers of each of these networks were modified and adapted to the dimensions of our chemical images (256 × 256 pixels × 106 channels). Figure 3a depicts the modifications employed for one of the architectures, the LeNet5 network. A fully connected layer replaces the third convolutional layer, and the first two convolutional layers have a variable number of filters. In all the networks the convolution filters used were of dimension 5 × 5, and pooling windows were size 2 × 2, with a stride of 1 in each spatial dimension. Convolution layer kernel weights and biases were initialized using the *keras* built-in uniform He-initializer, and activated using the ReLu function, with training initially for 500 epochs. The data was again split by patient in a random, stratified manner into training, validation and test sets in a 60:20:20 ratio. Model performance was expressed in terms of the receiver-operator characteristic area-under-curve of each test set, and this splitting, training and testing was performed 5 times. Following the emergence of a leading model (LeNet5), the effects of batch size on the performance of the modified version of LeNet5 were examined. Batch sizes of 8, 16, 20, and 32 were examined for prediction of recurrence, along with batch normalization (BN) between convolution and pooling layers. A *k*-fold train test split with 5 folds, with a constant random seed, was used to form training and testing sets from here on. These models, and all subsequent models (unless otherwise stated) were trained for 150–200 epochs. For regularization, if the training loss did not decrease over 60 epochs, training was halted using the *keras* callback early stopping option, and the model parameters at the lowest loss value were saved. The average test ROC AUC over the 5 folds is reported.

A 3D filter is a cube which convolves over the entire image resulting a 3D feature map of the same or of a slightly reduced size. This introduces two major sources of RAM drainage. Firstly, layers in a 3D-DL-CNN comprise of several filters, each of which outputs several 3D feature maps which must then be input into the layer following, all of which absorbs substantial amounts of memory. Secondly, the number of learnable parameters in a 3D convolution layer is increased significantly. Here the implementation of 3D-CNN layers proved infeasible owing to RAM drainage and this aspect was not explored further.

As an alternative depth-wise separable convolution (DSC) layers were employed, replacing the 2D convolution layers in the champion network emerging from approach (c) to allow deeper mining of the spectral information. Additionally, other network architectures built from DSC layers were trialed for this task, with modifications made to result in lighter models.

Approach (a) trained a model solely on spectral data, and examined one subset of data at a time. Approach (b) clearly does not leverage the rich spectral information within the chemical image, but does allow mining of the spatial distribution of features within the image. Similarly in approach (c) each convolutional filter is passed over all channels individually, and the resulting 3D feature map is flattened to a single channel array by vector addition such that a single channel input is provided to the remaining layers.

**Fig. 4:**
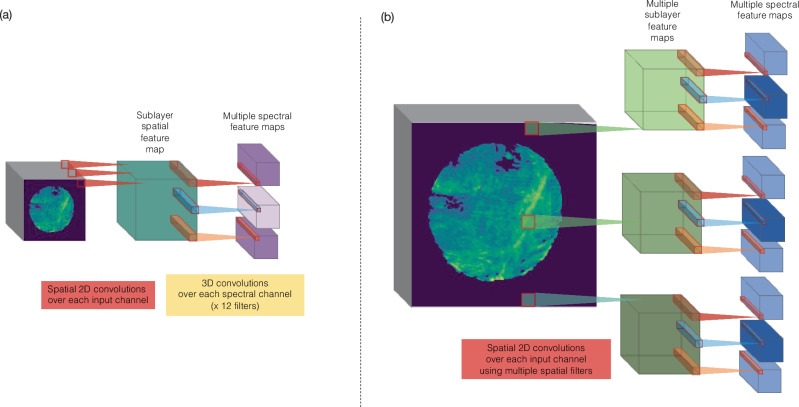
Operation of depth wise separable convolutions. Visualization of the operation of (**a**) Type 1 and (**b**) Type 2 depthwise separable convolution operation employed in this work.

Approach (d) on the other hand does allow mining of the spectral information and spatial information in which the 3D feature map is not flattened by simple vector addition, but instead is used as a sublayer input for the convolution in the spectral dimension which follows, by passing a 1D-convolution kernel with learnable weights over the spectral information, or image channels (Fig. 4). We have employed two implementations of this approach, which we here refer to as *Type 1* (Fig. 4a) and *Type 2* (Fig. 4b).

**Fig. 5:**
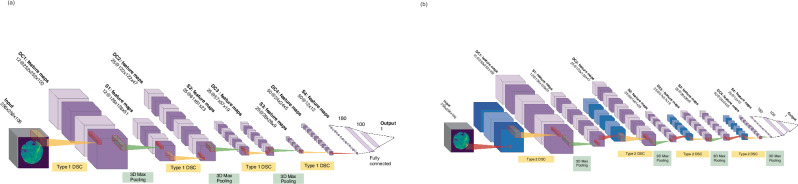
Depth-wise separable CNN architectures for prediction of treatment outcomes with chemical imaging data. **a** Depthwise separable convolutional neural network with Type 1 separable convolutions. Depthwise separable convolutional (DSC) operations are shown in yellow with max-pooling operations shown in green. **b** Depthwise separable convolutional neural network with Type 2 separable convolutions. Depthwise separable convolutional (DSC) operations are shown in yellow with max-pooling operations shown in green.

In *Type 1*, a single filter (x-y) is passed over the spatial image dimensions as a standard 2D filter. At this step, several 1D filters are passed over the spectral dimension of this feature map, to produce several 3D feature maps, similar to the operation of a standard 3D convolution layer. This layer will then mine mostly spectral information with some spatial components. Type 1 DSCs are implemented as shown within the network in Fig. 5a.

In the *Type 2* approach, several (x-y) filters are passed over the input chemical image, producing sublayer feature maps. Again, 1D filters are then passed over these filter maps in the spectral (z)-dimension. This results in rapidly increasing amounts of high dimensional feature maps and provides an architecture which provides deeper mining of both the spatial and spectral information within the image. Type 2 DSCs are implemented as shown within the network in Fig. 5b.

Ultimately both of these approaches allowed us to increase the degree to which the spectral information was mined by our DL algorithms using various approaches:

1. Initially, a single adjustment was made to the modified LeNet5 architecture, whereby the input layer 2D convolutions were replaced with a separable convolution layer (Model 1). Following that, Models 2, 3, and 4 were constructed using Type 1 depthwise-separable convolution layers (Fig. 5a). These models varied in the rate at which the spectral dimension was reduced, by varying pooling types and window size, as well as the number of feature maps produced per layer. The first layers of each model were the same, consisting of a depthwise-separable convolution (DSC) with 12 individual filters of dimension 5 × 5 × 5 pixels, followed by 3D Max pooling with dimension 2 × 2 × 2 pixels, another DSC layer with 25 individual 5 × 5 × 5 pixel filters, and a 3D Max pooling layer.
  Models 2 and 3 had a third DSC with 25 individual 5 × 5 × 5 pixel filters. The latter half of Model 2 was made up of a 3D max pooling layer, a DSC layer with 50 individual 5 × 5 × 5 pixel filters and a second 3D max pooling layer. Model 3 had a 1D average pooling layer following the third DSC, collapsing the spectral dimension of the feature maps entirely, and a 2D max pooling layer (2 × 2) to condense the spatial dimension. Model 4 had one DSC with 50 individual 5 × 5 × 5 pixel filters following the 2nd pooling layer, after which a 3D global average pooling layer was employed. For each of these models, the remaining features were flattened to one dimension, which were fed into a 180 node fully connected layer, followed by a fully connected layer of dimension 100, followed by the output layer that had dimension 1, and which utilized the sigmoid activation function. Each model was trained using a binary cross-entropy loss function and an adam optimizer. All models were trained with accuracy used as the metric of performance, with weighting for class imbalance.
2. Model 5 was constructed using Type 2 depthwise-separable convolution layers, as shown in Fig. 5b. The first layer of Model 5 is a type 2 DSCNN made up of 5 × 3 filters, with 5 of dimension 5 × 5 × 1 pixels, followed by 3 of dimension 1 × 1 × 5 pixels, resulting in 15 × 3D feature maps used to input to the subsequent 3D max pooling layer, with dimension 2 × 2 × 2 pixels. The next type 2 DSC layer was made up of 5 × 5 pixel filters, of the same dimensions, and second 3D max pooling layer. This was repeated for the 3rd DSC and pooling layers. The final type 2 DSC layer consisted of 5 × 10 pixel filters, resulting in 50 feature maps to be pooled using a 3D max pooling layer with window size 2 × 2 × 5 pixels. The resulting feature maps were flattened and fed into two fully connected layers of 180 and 100 nodes. The output was from a single node activated by a sigmoid function.

To further combat RAM issues, a model designed to reduce the size of feature maps was then designed. The input was reduced using a 3D average pooling window of size 4 × 4 × 4 pixels, before DSC type 1 convolutional filters of greater dimension (8 × 8 × 8 pixels and 3 × 3 × 5 pixels) were applied. After two DSC layers and pooling layers, the spectral dimension was reduced such that a 2D convolution layer and max pooling layer was placed before the feature maps were flattened and fed into the fully connected layers and class was output.

## Results

### Hybrid ML (PLSDA)—TL modeling

Models developed using an image segmentation—traditional ML pipeline with PLSDA were overall poor at predicting BCR, with no image segment or spectral subset observed as a prominent marker of recurrence.

Similarly, using the primary PLS-fed transfer learning approach, no model was successful in classifying BC recurrence (BCR). The performances of CNN models trained for classification of BCR using this dataset are listed in Table 2.

**Table 2:** Performance of transfer learning approach with image dimensionality reduction via PLSDA

DL network	ROC AUC
VGG16	0.500 ± 0.000
Xception	0.524 ± 0.060
InceptionV3	0.499 ± 0.001
DenseNet121	0.500 ± 0.000

### Modified 2-D CNNs

**Fig. 6:**
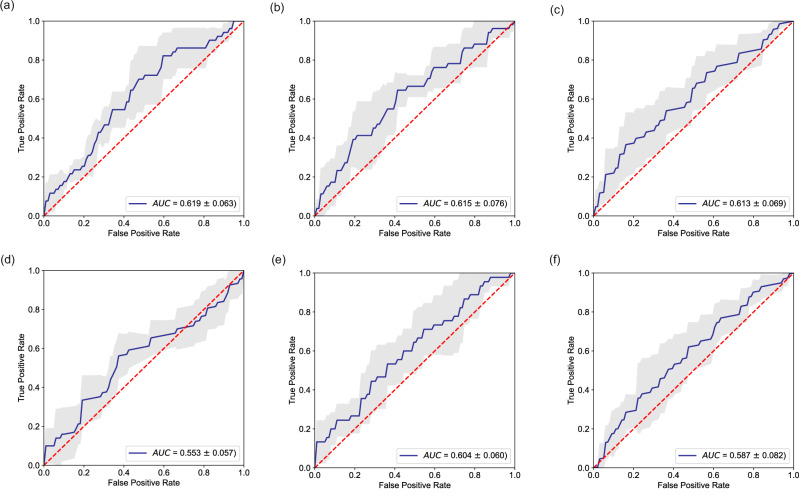
Sample receiver operator characteristics for DSCNN models. **a** Model 2 (with batch normalization and a batch size of 8); (**b**) Model 2 (with batch normalization and a batch size of 16); (**c**) Model 3; (**d**) Model 4; (**e**) Model 5; (**f**) Model 6. The reader is referred to the main text to obtain details on the modeling parameters for each model variant.

The performances of the networks in classifying both BCR and survival after therapy BC are presented in Table 3, and model ROCs across all modeling instances are visualized in Figs. 3b and 6. InceptionV3 and LeNet5 were capable of classifying recurrence with ROC-AUCs of 0.577 ± 0.054 and 0.639 ± 0.081, respectively (an average receiver operator characteristic (ROC) for the LeNet5 architecture is depicted in Fig. 4b). The LeNet5 architecture has ~16 × 10^6^ learnable parameters.

**Table 3:** Performance of modified 2D CNN networks in predicting BC recurrence

DL network	ROC AUC
VGG16	0.515 ± 0.030
Xception	0.524 ± 0.028
InceptionV3	0.577 ± 0.054
DenseNet121	0.536 ± 0.075
AlexNet	0.483 ± 0.023
LeNet5	**0.639** ± **0.081**

The value in bold denotes the highest classification performance achieved across all network architectures.

As the modified LeNet5 architecture was found to be the champion model here (Fig. 3b), an interrogation of the effect of batch size during training was considered, for classifying both BCR and early (up to 7-year) survival after therapy for BC. These results are shown in Table 4.

**Table 4:** Modified LeNet 5 model performance with classification task and batch normalization approach

Batch size	Class label	ROC AUC
8	BCR survival	0.576 ± 0.080
8	BCR survival	**0.680** ± **0.060**
20	BCR survival	0.639 ± 0.081
8	BCR	0.600 ± 0.149
16	BCR	**0.607** **±** **0.094**
32	BCR	0.572 ± 0.063

The values in bold highlight the maximal classification performance observed.

### Depthwise-separable CNNs

After substituting the first layer of the modified LeNet5 model with a 3D separable convolution layer, the average ROC AUC was 0.641 ± 0.095, similar to the purely 2D convolutional neural network. Models 2–6, which were built entirely using DSC layers produced a drop in performance. Tables 5 and 6 depict the overall results of these investigations, demonstrating the average receiver operator characteristic curves over 5 separate executions of each model. Figure 6 depicts the typical ROC profiles and AUCs observed for each of these models.

**Table 5:** Performance of modified LeNet5 model architecture with a separable convolutional layer as the first layer, and a Type 1 DSCN model with 4 convolutional layers for prediction of BCR and BC survival

Model	Layer type	Depth	No. params	Batch size	Class label	ROC AUC
Model 1	S-2D CONV	4	16.7 × 10^6^	8	BCR survival	**0.641** ± **0.095**
Model 1	S-2D CONV	4	16.7 × 10^6^	8	BCR	0.600 ± 0.122
Model 2	DSC1	6	1.3 × 10^6^	8	BCR survival	0.608 ± 0.068
Model 2	DSC1	6	1.3 × 10^6^	8	BCR	**0.628** ± **0.098**

Here, depth is number of layers with learnable parameters, excluding the output single node layer.

Values in bold denote the highest classification performance achieved for each network architecture and classification task.

**Table 6:** Performance of Type 1 and Type 2 depthwise separable convolution models for prediction of BCR

Model	Layer type	Depth	No. params	Batch size	Class label	ROC AUC
Model 3	DSC1	5	3.5 × 10^6^	8	BCR	0.521 ± 0.063
Model 4	DSC1	5	3.2 × 10^4^	8	BCR	0.557 ± 0.068
Model 5	DSC2	6	1.3 × 10^6^	8	BCR	0.588 ± 0.131
Model 6	POOL-DSC1	5	1.0 × 10^5^	16	BCR	0.546 ± 0.065

Here, depth is number of layers with learnable parameters, excluding the output single node layer.

In 2D convolutional layers, the image channels (in this case the IR spectral dimension) collapses to a single value per pixel by vector addition. In Model 1, this was done in a learnable manner, by way of a separable convolutional layer. This did not have a significant deleterious effect on performance. Following this, maintaining the spectral dimension as a minable aspect of the data deeper in the networks was a focus of network design. Even the use of 3D depthwise-separable convolutions over 3D convolutions did not completely eradicate the RAM constraints associated with this type of network. While the number of learnable parameters within each of the DSC models were orders of magnitude lower than both the 2D CNNs, maintaining several 3D feature maps of a batch of samples in computer memory during training presented a significant obstacle. Batch size could not be increased beyond 8 in models 2–5, which would have had an affect on the variability of the model performances.

Each of the 3D-DSCNN models were smaller in terms of parameters, with model 3 being larger than model 2 (~3.5 × 10^6^ versus 1.3 × 10^6^ parameters), and Model 4 being the smallest at ~ 32 × 10^4^ parameters. Model 5 with type 2 DSC had ~ 1 × 10^6^ parameters, and the model designed for greater dimensionality reduction, Model 6, had ~1 × 10^5^ parameters. Each of these models were trained using batches of 8 samples, except Model 6 which was trained using a batch size of 16.

Of the type 1 DSCNN models, Model 3, despite being the largest DSCNN model type, exhibited the lowest performance. Model 4, the smallest model overall, had 3 DSC layers. Notably this model had the same depth as model 3, but exhibited superior performance. Here, instead of flattening the final feature maps for input to fully connected layers, which uses a lot of RAM, global average pooling layer was used to reduce these to single values. Model 2 was the deepest of the type 1 DSC models, but not the most complex, and was the best performing model by a slight margin.

Using the type 2 DSCNN, Model 5, had a poorer performance, and took much longer to train, though this model was the only type to mine both the spatial and spectral dimensions to an equal extent. Reducing both spatial and spectral dimensions to produce more manageably sized feature maps was the goal of implementation of Model 6. This model had slightly poorer performance, and also had fewer learnable parameters than the best performing type 1 DSCNN model, Model 2.

## Discussion

Chemical imaging in histopathology is relatively well established as an approach for the development of label-free diagnostic pipelines for cancer^22,25^, with a recent focus being applied on the development of methods for prediction of post-treatment response^13^. Typically, pipeline development in the field has employed image segmentation followed by the application of machine learning approaches on spatially binned spectral data, for classification of disease type. This is a time consuming process requiring pooling or separation of groups of spectra, or intense dimensionality reduction. The central hypothesis of this study is that the use of deep learning approaches on chemical image data, where spectral information is not separated from its spatial context, will lead to improved performance in predicting disease recurrence, where spatial context within the tumor microenvironment is known to be a contributory factor^49–51^.

Here the performances of ML and hybrid ML-DL-TL models validate this hypothesis, suggesting that a more biochemically holistic image of the tissue sample is required. As clusters of FTIR spectra correspond loosely to subtypes of tissue, of which none individually could be used as a predictor of recurrence here, this result overall suggests that separating the spectral and spatial components of chemical imaging datasets is insufficient for the development of models of treatment outcome. However while it is acknowledged that a from-scratch approach to training of models with PLS embedded images might have provided improved performance, this aspect was not explored.

While the development of deep learning models with image data from scratch requires the use of very large datasets of the order of >10^6^ images, it has been demonstrated in many instances that robust models which do not overfit can be developed using small datasets^52,53^. In the context of the present study, it is firstly notable that, of the widely cited image based 2D-CNN architectures trained from scratch on chemical images, the shallowest, LeNet5, was the champion model, with Inception-V3 as the next best model. As the dataset used in this study is small compared to the datasets typically used for training of DL models, which typically comprise millions of images, the performance of the network was quite dependent on the training batch size, resulting in wide measures of uncertainty. Additionally, the sample set is imbalanced with respect to recurrence, with samples mainly confined to the negative class. This was mitigated to an extent during training by weighting samples of the recurrence class, but this imbalance still affected the training loss gradient. Overall, after varying the batch size from 8 to 16 to 32, a slight increase in average ROC AUC value was associated with smaller batch sizes, but a slight reduction in uncertainty was associated with larger batch sizes. The task re-definition also made a small difference to the predictive performance of the models, where models predicting BC survival (with a batch size 8 and batch normalization) were most successful, having an average ROC AUC of 0.68 ± 0.06. This hints at the possible benefit of re-examining the definition of the task, perhaps excluding those patients with mortality by causes other than breast cancer prior to any potential recurrence event.

Again, we observed that while the batch training performance of the 2D-CNN model did not vary substantially, the result of this part of the analysis confirms that the coupling of spectral and spatial information within DL models leads overall to superior predictive performance for BCR, approaching that of validated proteomic and genomic tests in this space.

This result is again echoed in the continuation work which looked to develop depthwise-separable CNNs for more effective mining of the spectral information within FTIR chemical images. Overall that analysis suggests that an optimal depth and complexity exists for network models for this application, below which model performance is reduced. Type 1 DSCNN models appear to be superior to Type 2 models for this application, with Model 2 producing a ROC AUC performance for prediction of BCR of 0.628 ± 0.095. This model was comparable in performance to the modified 2D-DCNN LeNet5 architecture (with ROC AUC of 0.639 ± 0.081) and while it was the deepest of the Type 1 models, it was not the most complex. Therefore, the coupling between spectral and spatial information offered by chemical images is best leveraged through relatively shallow DL networks for prediction of future treatment outcomes, with no loss of performance seen when layer outputs are pooled harshly.

For contextualization of the performance of the networks designed in this work, equivalent studies in digital pathology provide a useful reference. Within the field of digital pathology substantial advances have been made towards the use of digital images acquired from standard H&E specimens within automated pathological support systems^40,54^. Within the breast cancer field the approach has successfully demonstrated the capability to classify disease grade^55,56^, subtype^56,57^ and for automated image segmentation, including the identification of the location and distribution of tumor infiltrating lymphocytes, which is itself an important prognostic indicator^49,58–60^. More recently the field has demonstrated potential to classify survival probability^61,62^ and risk of recurrence using OncoType-DX genetic risk scores as ground truth^63^. In particular Whitney et al.^64^ have demonstrated the capability of digital pathological metrics to classify OncoType-DX scores at low versus intermediate/high levels with ROC AUC of 0.58 and low+intermediate versus high with a ROC AUC of 0.6. Similarly Howard et al. have classified high OncoType DX scores using a purely image-based model with ROC AUC of 0.797 (95% CI 0.68–0.901) and a model incorporating a clinical nomogram with a ROC AUC of 0.814 (0.709 to 0.901)^65^. As we have developed models for prediction of recurrence, including patients across all risk profiles, these studies are not directly comparable with our own, though the work of Whitney et al. does demonstrate a similar level of performance to our own for a similar predictive objective.

As genetic alternations of tissue precede morphological changes that are diagnostic or prognostic^66,67^, downstream chemical changes to tissue owing to alterations in transcription and protein translation will be visible spectroscopically prior to their morphological manifestation. Critically, H&E based digital pathology only assesses spatial and morphological metrics for diagnostic and prognostic purposes, while chemical imaging measures the intrinsic biochemistry of tissues spatially. Our approach develops models whose performance exceeds those of purely AI-based histomorphometric approaches, suggesting the existence of spatial chemical signatures which may offer a performance advantage for prediction of recurrence. Practically this approach may also have application in a number of contexts, as (1) a first stage triage for recurrence prediction, or (2) in surgery in a similar fashion to that seen recently with stimulated Raman imaging^17^, allowing for near real-time decision making in a surgical context. Ultimately this label-free all digital approach may hold promise in improving estimates of recurrence within breast cancer, particularly for those patients with low risk of recurrence.

Ultimately, while bulk molecular assays of pathological samples do provide an avenue for the development of validated prognostic tests for cancer treatment outcome, their explanatory utility for cell function is limited given the absence of spatial context. Chemical imaging, and similar deep spatial biochemical profiling technologies do provide a novel opportunity for the development of prognostic technologies that also provide insights into spatial context in a label-free manner^24^. Recent investigations have demonstrated that mainstream two-dimensional convolutional networks do not effectively mine the rich, exquisitely sensitive, molecular information within these images, rather mining the spatial information within the images^68^.

As exemplified by this study, analytical pipelines can be developed that have prognostic value in early stage breast cancer, which have demonstrated performance in prediction of all breast cancer recurrence events (ROC AUC of approximately 0.64) which aligns well with bulk molecular tests and AI-detected histomorphometric tests in this space. Our work demonstrates that appropriately designed convolutional neural networks are capable of learning the spectral and spatial characteristics within breast tissue pathology to provide robust prognostics in a strongly heterogeneous context. Importantly this study demonstrates for the first time that vibrational spectra of histopathological samples, and their spatial context, are signatures with prognostic value which may have potential for more widespread use in objective, label-free all-digital chemical imaging driven histopathological prognostics. Future work will examine the potential of this pipeline for prediction of post-treatment survival probability, and other treatment endpoints in BC and other cancers, for further enhancement of the power of the technology.

## Data Availability

Data associated with this work may be shared with interested parties upon reasonable request.

## Code availability

Code associated with this work may be shared with interested parties upon reasonable request.
